# Utility of baseline, interim and end-of-treatment ^18^F-FDG PET/CT in extranodal natural killer/T-cell lymphoma patients treated with L-asparaginase/pegaspargase

**DOI:** 10.1038/srep41057

**Published:** 2017-01-24

**Authors:** Yu Chang, Xiaorui Fu, Zhenchang Sun, Xinli Xie, Ruihua Wang, Zhaoming Li, Xudong Zhang, Guangyao Sheng, Mingzhi Zhang

**Affiliations:** 1 Department of Oncology, the First Affiliated Hospital of Zhengzhou University, No. 1 Jianshe East Road, Zhengzhou, 450052, China; 2 Department of nuclear medicine, the First Affiliated Hospital of Zhengzhou University, No. 1 Jianshe East Road, Zhengzhou, 450052, China; 3 Department of Pediatrics, the First Affiliated Hospital of Zhengzhou University, No. 1 Jianshe East Road, Zhengzhou, 450052, China

## Abstract

Positron emission tomography-computed tomography (PET/CT) is widely used for initial staging and monitoring treatment responses in Hodgkin and diffuse large B-cell lymphoma. However, its prognostic value in extranodal natural killer (NK)/T-cell lymphoma (ENKL) remains unclear. Here, we conducted a retrospective study to determine the impact of PET/CT in ENKL. Fifty-two patients newly diagnosed with ENKL were enrolled. Baseline maximum standardized uptake values (SUVmax), whole-body metabolic tumor volume (WBMTV) and whole-body total lesion glycolysis (WBTLG) were recorded. Additionally, interim PET/CT (I-PET) and end-of-treatment PET/CT (E-PET) results were scored using a 5-point scale. Patients were divided into groups using baseline parameter cut-off values; significant differences were found in overall survival (OS) and progression-free survival (PFS) between the high and low WBMTV and WBTLG groups and in OS between the two SUVmax groups. Positive I-PET and E-PET results predicted inferior PFS and OS. A multivariate analysis showed that baseline WBTLG, I-PET and E-PET results were associated with PFS and OS, and baseline SUVmax was an independent predictor of OS. Thus, baseline WBTLG, I-PET and E-PET results are good predictors of PFS and OS in ENKL patients who received L-asparaginase/pegaspargase in their first-line treatment, and baseline SUVmax is a valuable tool for assessing OS.

Extranodal natural killer/T-cell lymphoma, nasal type (ENKL) has been recognized as a distinct clinicopathologic type with an aggressive clinical course and a remarkable geographical prevalence in Asia and South America^1^,^2^. It is also the most common peripheral T-cell lymphoma (PTCL) in Asia, accounting for 22.4% of PTCL^3^,^4^. The prognosis of ENKL is poorer than that of B-cell lymphoma. The reported 5-year overall survival (OS) rate is 32%, and the median survival time is approximately 8 months^5^,^6^. To date, the optimal therapy remains unestablished. However, L-asparaginase/pegaspargase-based combination chemotherapy with or without radiotherapy (RT) has shown promise in this context.

During the last decade, many studies have shown the superior accuracy and sensitivity of ^18^F-fluorodeoxyglucose positron emission tomography-computed tomography (PET/CT) over CT^7^,^8^. Mounting evidence supports the central role of PET/CT in staging and assessing treatment response in malignant lymphoma, especially in Hodgkin lymphoma (HL), diffuse large B-cell lymphoma (DLBCL) and follicular lymphoma (FL)^9^,^10^,^11^,^12^. Meanwhile, recent studies have confirmed the beneficial role of PET/CT in detecting bone marrow infiltration in HL and DLBCL, even obviating the need for biopsy^13^,^14^. Currently, few reports are available on the prognostic value of PET/CT in ENKL. Only one has discussed PET/CT in all stages, including baseline PET/CT (B-PET), interim PET/CT (I-PET) and end-of-treatment PET/CT (E-PET)^15^. Furthermore, the regimens used in these limited previous studies included CHOP, CHOP-like or L-asparaginase/pegaspargase-based regimens, and the criteria for interpreting the PET/CT results also varied^16^,^17^,^18^. Therefore, some concluded that PET/CT has prognostic value in ENKL^15^, while others did not^19^. Thus, the prognostic value of PET/CT in this aggressive malignancy remains controversial. How to optimize the use of PET/CT in ENKL to identify individuals with worse prognosis is also an issue that merits research.

Therefore, in this study, we investigated the prognostic value of baseline, interim and end-of-treatment PET/CT results in ENKL in a single-center study.

## Patients and Methods

### Patient Selection

In all, 52 patients newly diagnosed with ENKL and treated at the Lymphoma Diagnosis and Treatment Center of Henan Province were enrolled from April 2011 to December 2015 in this retrospective study. All patients had a pathological diagnosis of ENKL according to the World Health Organization lymphoma classification criteria^20^, as determined by pathologists. All included patients had undergone at least one of the following three PET/CT scans: B-PET, I-PET (after 2 to 4 cycles of chemotherapy) and E-PET (after first-line therapy). The lymphoma stage was evaluated by the Ann Arbor staging system.

During the diagnosis, mid-treatment and after the completion of the first-line regimen, the patients underwent routine evaluations, including a physical examination, blood routine tests, a blood biochemical examination, bone marrow aspiration and a biopsy, CT or MRI if necessary. Patients with central nervous system involvement were excluded.

This study was approved by the ethics committee of the First Affiliated Hospital of Zhengzhou University. Informed consent for the collection of medical information was obtained from all patients. All procedures performed in the study were in accordance with the ethical standards of the institutional research committee.

### PET/CT Protocol and Image Analysis

The PET/CT scans were acquired on a dedicated PET/CT scanner (Siemens Biograph 64 Truepoint PET/CT, Germany). Patients fasted overnight before the injection of 3.7–4.4 MBq/kg of ^18^F-FDG. Blood glucose (<6 mmol/L) was checked before tracer injection. PET/CT scans were performed after resting for 60 ± 15 minutes. A CT scan was obtained initially with a voltage of 120 kV, a current intensity of 100–250 mA, a tube rotation of 0.8 s, and a section thickness of 5 mm. PET scans were obtained at 2.5 min per bed position.

The PET and CT images were then reviewed on a dedicated workstation (Syngo MMWO VE36A, Siemens, Germany). Images were retrospectively reviewed by two experienced nuclear medicine physicians. The B-PET scans were evaluated by maximum standardized uptake value (SUVmax), whole-body metabolic tumour volume (WBMTV) and whole-body total lesion glycolysis (WBTLG). Dedicated software (Syngo TrueD) estimated the SUVmax and MTV data using an isocontour threshold method based on 40% of the SUVmax. The baseline SUVmax of all lesions was recorded, and the highest value was considered the SUVmax of the patient. For the I-PET and E-PET scans, we recorded the highest SUVmax of all the residual lesions in the scan regardless of the index lesions. If the lesion was no longer observed after treatment, a region of interest was drawn in the area of the same lesion in the B-PET scan. The TLG was then calculated as the product of the MTV and the average SUV. The WBMTV and WBTLG were obtained by summing the MTV and TLG of all lesions, respectively. The reduction in SUVmax (ΔSUVmax) between the B-PET and I-PET scans was analyzed for the patients with B-PET scans. The percentage ofΔSUVmax was calculated as follows: ΔSUVmax (%) = 100 × [SUVmax (baseline) − SUVmax (I-PET)]/SUVmax(baseline). The I-PET and E-PET scans were interpreted using a five-point scale (5-PS) based on the Deauville criteria^21^. The 5-PS scores the most intense uptake in a site of initial disease as follows: 1, no uptake; 2, uptake ≤ that of the mediastinum; 3, uptake > that of the mediastinum but ≤ that of the liver; 4, uptake moderately greater than that of the liver; 5, uptake markedly greater than that of the liver and/or the presence of new lesions; and X, new areas of uptake were unlikely to be related to lymphoma. Scores of 1 to 3 were considered negative, and scores of 4 and 5 were considered positive^22^.

### Statistical Analysis

The receiver operating characteristic (ROC) curve and the area under the ROC curve (AUC) analysis showed optimal cut-off values for SUVmax. Differences in SUVmax between the progression and progression-free groups, as well as those between the death and the survival groups, were assessed using the Mann-Whitney U-test. The Pearson χ^2^ test and Fisher exact tests were used to analyze the relationships between the PET/CT results and clinical variables [gender, age, B symptoms, Eastern Cooperative Oncology Group (ECOG) performance status, serum lactate dehydrogenase (LDH) level, International Prognostic Index (IPI), Korean prognostic index (KPI), Ann Arbor staging and lesion site]. OS and progression-free survival (PFS) were chosen as endpoints to evaluate the prognostic value of PET/CT. OS was defined as the period from diagnosis to death. PFS was defined as the period from diagnosis to disease relapse, progression or death from any cause. The log-rank test and the Kaplan–Meier method were used for a univariate survival analysis. A multivariate Cox proportional hazards model was used to identify the potential independent effects of clinical variables and PET/CT results. A *P* value less than 0.05 was considered statistically significant. The statistical software package SPSS 21.0 was used for statistical calculations.

## Results

### Patient Characteristics

We identified 52 patients (31 men, 21 women) with histologically proven ENKL at our institution. The median age was 40.5 years (range, 15–72 years). Patient characteristics are summarized in Table 1 (see supplementary information for more details). Based on lesion sites, 39 patients (75.0%) had upper aerodigestive tract (UAT) ENKL, and 13 patients (25.0%) had non-upper aerodigestive tract (NUAT) ENKL. The Skin, intestine and testes were the major extranasal sites. First-line treatments were a median of 3 cycles of a DDGP regimen (dexamethasone, cisplatin, gemcitabline and pegaspargase) or a modified SMILE regimen (dexamethasone, methotrexate, ifosfamide, L-asparaginase and etoposide) plus involved-field RT of 25 Gy for patients with stage I/II disease (n = 34), and 2–6 cycles of the same regimen for patients with stage III/IV disease (n = 18). One patient received autologous hematopoietic stem cell transplantation (ASCT) after the first-line treatment and had maintained disease-free survival to the end of the follow-up period. One patient was characterized by hemophagocytic lymphohistiocytosis (HLH) at initial presentation, and 2 patients subsequently developed HLH. Those 2 patients with HLH at the terminal phase of the disease died within 3 months. During a median follow-up period of 19 months (range, 5–55 months), the median OS for all 52 cases was 48 months. The 2-year PFS and OS rates for all patients were 50.0% and 70.0%, respectively.

### Baseline PET/CT

Of the 52 patients, 47 underwent B-PET. The median SUVmax at baseline was 13.7 (range, 3.3–28.8). The median SUVmax in patients with progression and progression-free disease were 16.3 (12.2–23.5) and 11.7 (9.0–16.9), respectively, and those in patients who died and survived were 19.0(15.5–27.3) and 12.2(9.0–16.8), respectively. A higher median SUVmax was associated with disease progression (*P* = 0.035) and a higher mortality rate (*P* = 0.001). There was no significant difference in SUVmax between patients with and without HLH [median, 23.5 (15.5–28.8) vs. 13.1(9.5–17.2), *P* = 0.050]. There was also no significant difference in SUVmax between UAT ENKL and NUAT ENKL patients [median, 12.3 (9.3–17.1) vs. 16.6 (14.0–22.4), *P* = 0.089].

**Figure 1:**
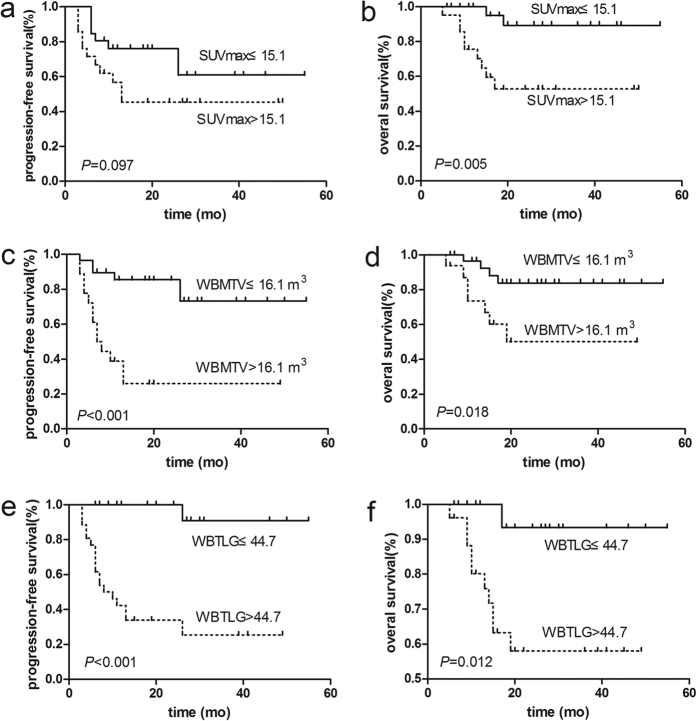
Kaplan-Meier survival curves according to the baseline SUVmax, WBMTV and WBTLG results. The baseline SUVmax result was associated with OS (**b**), but not PFS (**a**), as determined by the log-rank test. The baseline WBMTV and WBTLG results were associated with PFS (**c**,**e**) and OS (**d**,**f**).

The optimal SUVmax cut-off value on the ROC curve was 15.1 (sensitivity, 81.8%; specificity, 66.7%; AUC, 0.814). Clinical variables were then divided into two groups using this cut-off value. As shown in Table 2, a higher LDH level (*P* = 0.006) and a higher KPI index (*P* = 0.007) were both associated with a high SUVmax. In addition, B-PET parameters were similarly divided to generate Kaplan-Meier survival plots (Fig. 1). There was a significant difference in OS between patients with a high and low SUVmax (*P* = 0.005) but no difference in PFS (*P* = 0.097). The 2-year PFS and OS rates in high SUVmax group were 43% and 50%, respectively, and those in low SUVmax group were 56% and 88%, respectively.

The median WBMTV and WBTLG at baseline was 11.2 m^3^ (0.8–238.8 m^3^) and 46.4 (3.1–1858.1), respectively. The cut-off value of WBMTV and WBTLG on the ROC curve was 16.1 m^3^ (sensitivity, 63.2%; specificity, 85.7%; AUC, 0.799) and 44.7 (sensitivity, 89.5%; specificity, 67.9%; AUC, 0.803), respectively. When patients were separated into two WBMTV groups, the 2-year PFS rates (67% vs. 21%, *P* < 0.001, Fig. 1c) and OS rates (83% vs. 45%, *P* = 0.018, Fig. 1d) were significantly different between patients with a high and low WBMTV. Similarly, the 2-year PFS (83% vs. 26%, *P* < 0.001, Fig. 1e) and OS (93% vs. 52%, *P* = 0.012 Fig. 1f) differed significantly between patients with a high and low WBTLG, as determined by the log-rank test. As shown in Table 2, a high WBMTV and a high WBTLG were both associated with a higher IPI index (*P* = 0.034 and *P* = 0.002, respectively), an advanced Ann Arbor stage (*P* = 0.007 and *P* = 0.028, respectively) and NUAT ENKL (*P* = 0.002 and *P* = 0.033, respectively). Meanwhile, a high WBTLG was also associated with increased B symptom (*P* = 0.016) and a higher KPI (*P* = 0.020).

The predictive values of baseline SUVmax, WBMTV and WBTLG were calculated and are shown in Table 3. The negative predictive values (NPVs) of these parameters for PFS and OS were both higher than the positive predictive values (PPVs).

### Interim PET/CT

**Figure 2:**
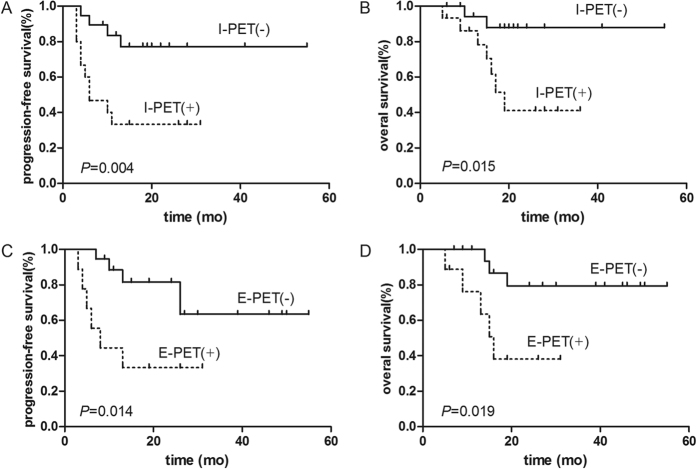
Kaplan-Meier survival curves according to the interim PET/CT and end-of-treatment PET/CT results. I-PET and E-PET results were both associated with PFS (**A** and **C**) and OS (**B** and **D**), as determined by the log-rank test.

In all, 34 of the 52 patients underwent I-PET (after 2–4 cycles of chemotherapy; median, 3 cycles), and 19 of them (55.9%) showed negative results. One patient’s I-PET was performed after 2 cycles of chemotherapy, 22 scans were performed after 3 cycles, and 11 were performed after 4 cycles. The median interval was 19.0 days (range, 13–31 days) after the completion of the previous chemotherapy. Patients with positive I-PET results had significantly inferior PFS (*P* = 0.004, Fig. 2A) and OS (*P* = 0.015, Fig. 2B) rates, as determined by the log-rank test. The 2-year PFS and OS rates were 75.0% and 86.0%, respectively, in patients with negative I-PET results, vs. 31.0% and 43.0%, respectively, in patients with positive results. A total of 10 (66.7%) patients with positive I-PET results showed treatment failure (progression or relapse), and 7 (46.7%) died during the follow-up period. Data regarding the PPV, NPV, sensitivity, specificity, positive likelihood ratio (PLR), negative likelihood ratio (NLR), and accuracy of the I-PET results in predicting PFS and OS are listed in Table 3.

Of the 34 patients with I-PET data, 29 also received B-PET scans. TheΔSUVmax percentages between the B-PET and I-PET results were calculated, and no significant difference was found in the median ΔSUVmax between the I-PET negative and positive groups (72.5% vs. 68.4%, *P* = 0.310).

### End-of-Treatment PET/CT

Twenty-eight patients underwent E-PET scans after completing their first-line treatment. The median time from completing the treatment to performing the B-PET scan was 31.5 days (range, 16–54 days). A total of 19 (67.9%) E-PET scans were interpreted as negative, and 9 (32.1%) were interpreted as positive. There was no significant association between the clinical variables and E-PET results (all *P* < 0.050) (Table 2). In accordance with the I-PET data, negative E-PET results were also associated with superior PFS (*P* = 0.014, Fig. 2C) and OS (*P* = 0.019, Fig. 2D). The 2-year PFS rates in the E-PET negative and positive groups were 62.0% and 32.0%, respectively, and the 2-year OS rates were 79.0% and 35.0%, respectively. A total of 6 (66.7%) patients with positive E-PET results had treatment failure, and 5 (55.6%) died during the follow-up period. Data regarding the PPV, NPV, sensitivity, specificity, PLR, NLR, and accuracy of the E-PET results in predicting PFS and OS are listed in Table 3.

Of the 28 patients with E-PET data, 12 also underwent I-PET scans. Comparing the I-PET and E-PET results, 2 patient with positive I-PET results showed negative E-PET results. While 1 patient with a negative I-PET result showed a positive E-PET result, the I-PET and E-PET results were consistent in 9 (75.0%) patients.

### Univariate and Multivariate Analyses for PFS and OS

The results of the univariate analysis of the clinical variables and PET parameters are shown in Tables 4, 5 and 6. Among patients who underwent B-PET scans, we found that the KPI (*P* = 0.015), baseline WBMTV (*P* = 0.018), baseline WBTLG (*P* = 0.012) and baseline SUVmax (*P* = 0.005) significantly influenced OS and that the first three also associated with inferior PFS (*P* = 0.036, *P* < 0.001 and *P* < 0.001, respectively). In terms of I-PET, there was a significant decrease in PFS in individuals who had higher KPIs (*P* = 0.019) or positive I-PET results (*P* = 0.004). The KPI (*P* = 0.032), Ann Arbor stage (*P* = 0.023) and I-PET results (*P* = 0.015) were associated with OS. Furthermore, when the I-PET scan was performed after 3 cycles of chemotherapy, it was a predictor of PFS (*P* = 0.047), while I-PET scans performed after 4 cycles of chemotherapy could not predict survival (*P* = 0.086 for PFS, *P* = 0.214 for OS). A significant predictor of unfavorable PFS and OS in patients with E-PET data was a positive E-PET result (*P* = 0.014 and *P* = 0.019, respectively).

A backward step multivariate Cox proportional hazards model was used to assess the prognostic value of the B-PET, I-PET and E-PET results for PFS and OS after controlling for potential independent prognostic factors (Table 7). The results showed that baseline WBTLG, I-PET and E-PET were independent predictors of PFS (*P* = 0.017, *P* = 0.005 and *P* = 0.025, respectively) and OS (*P* = 0.041, *P* = 0.032 and *P* = 0.034, respectively). Baseline SUVmax was an independent predictor of OS (*P* = 0.017).

## Discussion

The utility of PET/CT in malignant lymphoma has been continually studied. Apart from its role in initial staging and response evaluation, PET/CT may also be useful for the prediction of bone marrow involvement in HL and DLBCL. Almost all NK/T-cell lymphomas are FDG avid^23^. Nevertheless, the utility of PET/CT in ENKL during the baseline, interim and end-of-treatment periods remains unclear. To the best of our knowledge, this is the largest cohort of ENKL patients in a study assessing the prognostic value of PET/CT at three different time points.

The few current studies on B-PET in ENKL are mostly focused on diagnosis and staging^24^,^25^. In this study, we examined the prognostic value of baseline SUVmax, WBMTV and WBTLG. For SUVmax, our results demonstrated the relationship between the median baseline SUVmax and prognosis (progression and survival) of patients with ENKL. When patients were divided into two groups using the determined cut-off value, the results showed that a higher SUVmax was an independent predictor of OS but not a prognostic factor of PFS. Several studies have shown that baseline SUVmax could be a predictor of treatment response and survival rate in ENKL patients^15^,^26^. Kim CY *et al*. reported that a high baseline SUVmax was a significant predictor of PFS but not OS^16^. Potential explanations for this inconsistency with previous findings are described below. First, the SUVmax cut-off estimated by the ROC curve was different in our study, and the selection of different baseline SUVmax cut-offs may lead to different results regarding prognosis. Second, the small cohort size in these studies was a limitation on accuracy.

Meanwhile, baseline WBMTV and WBTLG were also analyzed in our study. WBMTV represents the extent of the tumor burden, and WBTLG represents a combined assessment of tumor volume and metabolism by B-PET/CT. The two indices have recently been considered important prognostic factors in DLBCL^27^. One study determined WBMTV and WBTLG based on pretreatment PET/CT scans in patients with ENKL^16^. The results showed that a high WBMTV was the best predictor of OS and PFS and that a high WBTLG was a significant predictors of PFS, which is not completely consistent with our results. Therefore, more well-designed studies are required.

In addition, the relationships of baseline SUVmax, WBMTV and WBTLG with clinical characteristics were also analyzed. The LDH level and KPI were associated with SUVmax, and the IPI, Ann Arbor stage and lesion sites were all related to WBMTV and WBTLG. Khong PL *et al*. reported that the SUVmax was relatively higher in intranasal cases than that in extranasal cases^17^. We assessed the baseline parameters in both UAT and NUAT ENKL, and we found no differences between the different SUVmax groups. Furthermore, our result showed that the presence or absence of HLH in ENKL patients did not influence ^18^F**-**FDG uptake. To the best of our knowledge, some studies have indicated that the baseline SUV might be related to the diagnosis and prognosis of patients with secondary HLH^28^,^29^; however, no studies have discussed ^18^F-FDG uptake of lymphoma patients with or without HLH.

Based on the IPI, a large proportion ENKL patients are categorized as low risk, although quite a few of these patients have a poor prognosis. Because of this limitation of the IPI in ENKL, a new prognostic grading system, i.e., the KPI, was established for ENKL^6^,^30^. This system shows better predictive discrimination than the IPI scoring system, as it can identify 4 risk groups with different survival outcomes based on 4 prognostic factors (B symptoms, staging, LDH level and regional lymph node involvement). However, this system has also been reported to have limitation in ENKL^31^,^32^. Therefore, both IPI and KPI were used in our study to grade patients by risk stratification. Although there are many known prognostic factors of ENKL, such as the IPI, KPI, B symptom, and EBV-DNA, there are still no effective early-stage clinical indices that can be used for the prognostic stratification of ENKL patients. Therefore, more prognostic factors need to be identified.

In terms of I-PET, DLBCL patients with negative I-PET results or a distinct reduction in FDG uptake in the interim stage generally have more favorable outcomes than those with positive I-PET results or non-distinct reduction in FDG uptake^33^,^34^. However, previous studies have shown conflicting results regarding whether I-PET offers useful clinical prognostic information for ENKL. Our data demonstrated that the I-PET results had good predictive prognostic value in ENKL patients. However, these findings are not consistent with those of Jiang C *et al*.^15^, whose study showed that I-PET results had little value in predicting survival. These opposite results may due to many factors. First, the chemotherapy regimens were different. The patients in our study received DDGP/modified SMILE, while the patients in their study received an LVP (L-asparaginase, vincristine and prednisone) regimen. Furthermore, in their study, most patients received RT prior to their I-PET scans, whereas the patients in our study received only chemotherapy prior to their I-PET scans. False-positive PET/CT results may persist longer after radiation therapy or chemoradiotherapy (2 to 3 months or longer) than after chemotherapy alone (2 weeks)^35^,^36^. Last, I-PET scans were analyzed by International Harmonization Project (IHP) criteria in their study, which differ from the Deauville criteria. Nevertheless, our result are consistent with other previous findings. In a prospective study, patients with ENKL were all treated with the SMILE protocol and received baseline, interim and end-of-treatment PET/CT scans. The analysis showed that I-PET results interpreted by the 5-PS was a significant independent predictor of both OS (*P* = 0.004) and PFS (*P* = 0.004). The estimated 2-year OS and PFS were 81% and 62%, respectively, in patients with a 5-PS score of 1–3, compared with 17% in patients with a 5-PS score of 4–5 (P < 0.001 and 0.001, respectively)^18^.

Furthermore, in our study, the NPVs of I-PET (78.9% and 89.5%) were higher than the PPVs (66.7% and 46.7%), suggesting that negative results mid-evaluation had a better predictive value for PFS and OS than positive results. Although there is no definitely evidence that adjusting treatment based on I-PET results could improve a prognosis, our findings show that I-PET results could be used as a chemosensitivity index to predict PFS and OS in ENKL. To resolve this issue, further research should be conducted with uniformly treated patients.

We noticed that similar to ours, the chemotherapy regimen used in the previous studies mentioned above was the SMILE regimen. Meanwhile, we also found that the regimens in most of the previous studies were confusing. Regimens which were used as an initial chemotherapy for ENKL in some studies included CHOP or CHOP-like regimens (e.g. EPOCH)^16^,^19^, which have nearly been confirmed to be invalid for ENKL. Therefore, it is predictable that the patients who received those treatment had worse prognoses. To date, L-asparaginase and gemcitabine-based combination chemotherapy have been highly successful in improving the treatment outcome of ENKL^37^,^38^,^39^,^40^. Pegaspargase is a PEGylated form of L-asparaginase that is less allergenic and has a longer half-life. Our prior work has demonstrated the significant efficacy and safety profile of the pegaspargase-based regimen (DDGP) in the treatment of newly diagnosed and relapsed/refractory ENKL^41^,^42^. In the present study, all patients received SMILE or DDGP to keep pace with international standards.

Some reports have shown that HL patients with negative E-PET results rarely experience relapse^43^,^44^. High NPVs have also been reported for patients with DLBCL^45^. Owing to the remarkably lower PPV, a biopsy may be required if further treatment is considered. In our study, the E-PET was an independent predictor of PFS and OS in ENKL, and this result was consistent with the findings of Jiang C^15^ and Kim SJ^46^. A study that used the SMILE protocol, mentioned above, also showed that the 5-PS score of the E-PET was a significant independent predictor of both PFS (*P* = 0.014) and OS (*P* = 0.018)^18^. Moreover, in the present study, the PPV of the E-PET for PFS was 66.7%, suggesting that patients with positive E-PET results had a high rate of progression or relapse and that those patients should be considered candidates for more aggressive treatments. Assessment with PET/CT could be used to guide decisions before high-dose chemotherapy and ASCT, but additional studies remain warranted.

Currently, there are many criteria for interpreting PET/CT scans. Previous studies have categorized patients by their positive or negative I-PET or E-PET results, as determined by the IHP criteria. However, this traditional binary scoring system has many disadvantages, particularly high subjectivity and a high false-positive rate. For interim and post-treatment imaging, the 5-PS was recommended for interpreting and reporting PET/CT results at the First International Workshop on PET in Lymphoma in Deauville, in 2009^22^. One study prospectively assessed 60 ENKL patients in terms of the prognostic value of I-PET results analyzed by 3 methods: the IHP criteria, the 5-PS, and ΔSUVmax. They found that the 5-PS had prognostic value^47^. Therefore, in our study, the 5-PS was used to describe I-PET and E-PET scans.

Although, compared with stand-alone CT, PET/CT has higher sensitivity, can better discriminate lymphoma and non-malignant lesions, and is an important tool for evaluating therapeutic responses, PET/CT also has limitations. First, PET-CT scans can present false-positives results. A study from JCO showed that the false-positive rate of I-PET was up to 87% and that the PPV was just 32% in DLBCL^48^. Treatment-induced inflammation and concomitant infections may lead to false-positive, and this risk is particularly pertinent in ENKL patients because of the common coexistence of rhinitis and neoplasm. This false-positive result may persist for up to 2 weeks after chemotherapy alone or for 2 to 3 months after radiation therapy or chemoradiotherapy. To minimize the frequency of these potentially confounding findings, PET/CT scans should not be performed for at least 3 weeks, preferably 6 to 8 weeks, after completion of therapy^35^. Next, the optimal time for performing I-PET scans remains undecided. Most evidence available is mainly concentrated on performing I-PET scans after 2–4 cycles of chemotherapy. PET/CT scans performed either too early or too late will not provide value in terms of early prognosis monitoring. Our results showed that I-PET scans performed after 3 cycles of chemotherapy may be a predictor of PFS. However, this finding must be confirmed by future studies with larger sample sizes. The problems mentioned above, especially those for interim PET/CT, cannot be avoided completely.

Compared with previous studies, our research has some advantages. First, all patients received pegaspargase/L-asparaginase-based combination chemotherapy as the first-line treatment, which was effective against ENKL. Only with effective treatments can imaging examinations, whether CT or PET/CT, fully play a role in assessing therapeutic effects. Furthermore, the 5P-S, which has been adopted as the preferred reporting method for interim and end-of-treatment results, was used to interpret results in our study; in addition, the 5-PS could represent different grades of untake more effectively. Similar to previous studies, this study has several potential limitations. It was conducted at a single center, and the number of patients involved was relatively small. Additionally, our study was retrospective. Therefore, prospective, large-scale, multicenter studies are required to confirm our findings.

## Conclusion

This study of ENKL patients who received L-asparaginase or pegaspargase as their first-line chemotherapy demonstrates that baseline WBTLG is a prognostic factor of PFS and OS and that baseline SUVmax is a prognostic factor of OS. This study also demonstrates the highly effective, independent prognostic value of interim and end-of-treatment PET/CT scans. Considering the poor prognosis of ENKL, more work needs to be done to confirm the prognostic value of PET/CT in the whole cohort of ENKL patients.

## Additional Information

**How to cite this article:** Chang, Y. *et al*. Utility of baseline, interim and end-of-treatment ^18^F-FDG PET/CT in extranodal natural killer/T-cell lymphoma patients treated with L-asparaginase/pegaspargase. *Sci. Rep.* **7**, 41057; doi: 10.1038/srep41057 (2017).

**Publisher's note:** Springer Nature remains neutral with regard to jurisdictional claims in published maps and institutional affiliations.

## Supplementary Material

Supplementary Dateset 1

## Figures and Tables

**Table 1:** Patient characteristics.

Characteristic	Total (%)
Sex (male)	31 (59.6)
Age (>60y)	6 (11.5)
B symptoms (Yes)	31 (59.6)
ECOG ≥ 2	15 (28.8)
LDH level > 245 U/L	23 (44.2)
IPI score	
0–1	38 (73.1)
2–5	14 (26.9)
KPI score	
0–2	34 (65.4)
3–4	18 (34.6)
Ann Arbor Stage	
I/II	34 (65.4)
III/IV	18 (34.6)
Lesions	
UAT	41 (78.8)
NUAT	11 (21.2)
HLH	
Yes	3 (5.8)
No	49 (94.2)

ECOG, Eastern Cooperative Oncology Group; IPI, International Prognostic Index; KPI, Korean prognostic index; UAT, upper aerodigestive tract; NUAT, non-upper aerodigestive tract; HLH, hemophagocytic lymphohistiocytosis.

**Table 2:** Data comparison of patients with baseline, interim and end-of-treatment PET/CT.

Characteristics	SUVmax		*p*	WBMTV		*p*	WBTLG		*p*	I-PET		*p*	E-PET		*p*
	≤15.1 n = 26	>15.1 n = 21		≤16.1 n = 31	>16.1 n = 16		≤44.7 n = 21	>44.7 n = 26		Neg n = 19	Pos n = 15		Neg n = 19	Pos n = 9	
Sex (M/F)	16/10	11/10	0.528	15/16	12/4	0.080	10/11	17/9	0.221	10/9	10/5	0.495	12/7	4/5	0.432
Age (≤60/>60)	23/3	20/1	0.763	28/3	15/1	1.000	19/2	24/2	1.000	18/1	12/3	0.299	17/2	8/1	1.000
B symptoms (No/Yes)	14/12	6/15	0.081	15/16	5/11	0.260	13/8	7/19	0.016	7/12	6/9	1.000	7/12	4/5	1.000
ECOG (<2/≥2)	18/8	16/5	0.596	23/8	11/5	0.959	18/3	16/10	0.065	17/2	11/4	0.370	13/6	5/4	0.677
LDH level (≤245/>245)	19/7	7/14	0.006	20/11	6/10	0.078	14/7	12/14	0.160	12/7	7/8	0.489	10/9	3/6	0.435
IPI score (0–1/2–5)	19/7	15/6	0.900	26/5	8/8	0.034	20/1	14/12	0.002	15/4	12/3	1.000	12/7	6/3	1.000
KPI score (0–2/3–4)	22/4	10/11	0.007	24/7	8/8	0.056	18/3	14/12	0.020	15/4	7/8	0.075	12/7	4/5	0.432
Ann Arbor Stage (I/II/III/IV)	19/7	10/11	0.074	24/7	6/10	0.007	17/4	13/13	0.028	13/6	10/5	1.000	12/7	4/5	0.432
Lesions (UAT/NUAT)	23/3	14/7	0.145	29/2	8/8	0.002	20/1	17/9	0.033	16/3	10/5	0.417	4/15	5/4	0.097

ECOG, Eastern Cooperative Oncology Group; IPI, International Prognostic Index; KPI, Korean prognostic index; UAT, upper aerodigestive tract; NUAT, non-upper aerodigestive tract; SUVmax, maximum standardized uptake value; WBMTV, whole-body metabolic tumour volume; WBTLG, whole-body total lesion glycolysis; I-PET, interim PET/CT; E-PET, end-of-treatment PET/CT; Neg, negative; Pos, positive.

**Table 3:** Predictive values of baseline, interim and end-of-treatment PET/CT.

	PPV (%)	NPV (%)	Se (%)	Sp (%)	PLR	NLR	ACC (%)
Baseline SUVmax (n = 47)							
PFS	52.4	69.2	57.9	64.3	1.62	0.65	61.7
OS	42.9	92.3	81.8	66.7	2.46	0.27	70.2
Baseline WBMTV (n = 47)							
PFS	75.0	77.4	63.2	85.7	4.42	0.43	76.6
OS	43.8	87.1	63.6	75.0	2.55	0.48	72.3
Baseline WBTLG (n = 47)							
PFS	65.4	90.5	89.5	67.9	2.78	0.16	76.6
OS	38.5	95.2	90.9	55.6	2.05	0.16	63.8
I-PET (n = 34)							
PFS	66.7	78.9	71.4	75.0	2.86	0.38	73.7
OS	46.7	89.5	77.8	68.0	2.43	0.33	70.6
E-PET (n = 28)							
PFS	66.7	73.7	54.5	82.4	3.10	0.55	71.4
OS	55.6	84.2	62.5	80.0	3.13	0.47	75.0

PPV, positive predictive value; NPV, negative predictive value; Se, sensitivity; Sp, specificity; PLR, positive likelihood ratio, NLR, negative likelihood ratio; ACC, accuracy; SUVmax, maximum standardized uptake value; WBMTV, whole-body metabolic tumour volume; WBTLG, whole-body total lesion glycolysis; I-PET, interim PET/CT; E-PET, end-of-treatment PET/CT; PFS, progression-free survival; OS, overall survival.

**Table 4:** Univariate analysis of factors in patients with baseline PET/CT (n = 47).

Risk Factor	PFS			OS		
	Mean (SE)	95% CI	*P*	Mean (SE)	95% CI	*P*
Sex						
Male	27.70 (4.36)	19.15–36.25	0.181	38.66 (3.62)	31.56–45.75	0.706
Female	40.06 (5.05)	30.16–49.97		44.81 (4.47)	36.05–53.57	
Age						
Age ≤ 60	34.42 (3.81)	26.97–41.88	0.813	—	—	0.243
Age > 60	28.25 (10.87)	6.93–49.67		—	—	
B symptoms						
No	41.03 (4.76)	31.69–50.36	0.085	48.17 (3.63)	41.06–55.28	0.150
Yes	26.20 (4.41)	17.56–34.85		35.44 (3.84)	27.91–42.97	
ECOG						
<2	31.85 (3.84)	24.34–39.37	0.776	40.28 (3.18)	34.05–46.51	0.673
≥2	32.77 (6.67)	19.69–45.85		41.39 (5.67)	30.28–52.49	
LDH level						
≤245 U/L	33.80 (4.23)	25.50–42.09	0.386	41.40 (3.39)	34.75–48.04	0.515
>245 U/L	31.50 (5.36)	21.00–41.99		41.09 (4.70)	31.88–50.31	
IPI score						
0–1	32.24 (3.79)	24.82–39.66	0.782	40.34 (3.17)	34.13–46.55	0.706
2–5	33.15 (6.57)	20.27–46.03		41.46 (5.66)	30.38–52.55	
KPI score						
0–2	39.09 (4.10)	31.05–47.13	0.036	48.44 (3.02)	42.53–54.35	0.015
3–4	16.33 (3.15)	10.17–22.50		21.54 (2.65)	16.36–26.73	
Ann Arbor Stage						
I/II	30.90 (3.55)	23.94–37.86	0.603	40.21 (2.64)	35.04–45.38	0.072
III/IV	32.65 (5.79)	21.31–43.99		36.71 (5.31)	26.30–47.12	
Baseline SUVmax						
≤15.1	39.10 (4.57)	30.15–48.05	0.097	50.99 (2.69)	45.72–56.25	0.005
>15.1	26.50 (4.63)	16.99–36.02		31.91 (4.47)	23.14–40.67	
Baseline WBMTV						
≤16.1	43.43 (3.79)	36.01–50.86	0.000	48.38 (3.05)	42.40–54.35	0.018
>16.1	17.50 (4.60)	8.48–26.52		30.71 (5.06)	20.80–40.63	
Baseline WBTLG						
≤44.7	49.93 (3.34)	43.38–56.48	0.000	52.47 (2.45)	47.67–57.26	0.012
>44.7	20.42 (4.02)	12.55–28.30		33.53 (3.78)	26.13–40.94	

ECOG, Eastern Cooperative Oncology Group; IPI, International Prognostic Index; KPI, Korean prognostic index; SUVmax, maximum standardized uptake value; WBMTV, whole-body metabolic tumour volume; WBTLG, whole-body total lesion glycolysis; PFS, progression-free survival; OS, overall survival.

**Table 5:** Univariate analysis of factors in patients with interim PET/CT (n = 34).

Risk Factor	PFS			OS		
	Mean (SE)	95% CI	*P*	Mean (SE)	95% CI	*P*
Sex						
Male	16.98 (2.50)	12.09–21.88	0.260	28.11 (2.66)	22.91–33.32	0.670
Female	40.37 (6.16)	28.30–52.45		43.83 (5.55)	32.96–54.71	
Age						
≤60	34.81 (4.49)	26.00–43.61	0.715	41.36 (4.02)	33.49–49.24	0.721
>60	16.50 (5.76)	5.21–27.79		31.00 (4.33)	22.51–39.49	
B symptoms						
No	43.92 (5.62)	32.90–54.94	0.072	48.53 (4.14)	40.46–56.70	0.112
Yes	21.74 (3.96)	13.98–29.49		29.21 (3.49)	22.38–36.04	
ECOG						
<2	20.95 (2.36)	16.32–25.58	0.684	25.54 (1.75)	22.11–28.97	0.954
≥2	30.67 (9.97)	11.13–50.21		41.33 (7.98)	25.70–56.97	
LDH level						
≤245 U/L	31.56 (3.63)	24.45–38.67	0.051	34.50 (2.86)	28.90–40.10	0.402
>245 U/L	24.75 (6.27)	12.46–37.04		37.71 (5.99)	25.96–49.46	
IPI score						
0–1	21.58 (2.37)	16.94–26.22	0.332	25.44 (1.75)	22.01–28.88	0.900
2–5	26.86 (9.24)	8.74–44.98		42.29 (7.68)	27.24–57.34	
KPI score						
0–2	41.36 (4.74)	32.07–50.66	0.019	48.22 (3.57)	41.22–55.23	0.032
3–4	17.75 (4.81)	8.32–27.18		25.38 (4.39)	16.77–33.98	
Ann Arbor Stage						
I/II	20.28 (2.21)	15.94–24.61	0.268	32.26 (1.97)	28.40–36.12	0.023
III/IV	28.36 (7.38)	13.89–42.83		31.07 (6.60)	18.14–44.01	
I–PET						
Negative	44.36 (4.70)	35.14–53.57	0.004	49.84 (3.42)	43.13–56.55	0.015
Positive	14.00 (3.16)	7.81–20.19		23.09 (3.29)	16.63–29.54	
I–PET3						
Negative (n = 13)	24.65 (2.17)	20.39–28.90	0.047	—	—	0.058
Positive (n = 9)	15.78 (3.71)	8.51–23.05		—	—	
I-PET4						
Negative (n = 6)	39.50 (9.01)	21.84–57.16	0.086	40.83 (8.20)	24.76–56.91	0.214
Positive (n = 5)	10.60 (4.69)	1.41–19.79		17.60 (3.34)	11.05–24.15	

ECOG, Eastern Cooperative Oncology Group; IPI, International Prognostic Index; KPI, Korean prognostic index; I-PET3, interim PET/CT carried out after 3 cycles of chemotherapy; I-PET4, interim PET/CT carried out after 4 cycles of chemotherapy; PFS, progression-free survival; OS, overall survival.

**Table 6:** Univariate analysis of factors in patients with end-of-treatment PET/CT (n = 28).

Risk Factor	PFS			OS		
	Mean (SE)	95% CI	*P*	Mean (SE)	95% CI	*P*
Sex						
Male	30.24 (5.39)	19.68–40.81	0.545	34.58 (4.81)	25.15–44.01	0.376
Female	39.21 (6.40)	26.66–51.76		46.05 (5.69)	34.89–57.20	
Age						
≤60	36.72 (4.71)	27.50–45.95	0.190	40.86 (4.39)	32.26–49.46	0.934
>60	20.33 (12.13)	0.00–44.11		38.67 (9.25)	20.53–56.80	
B symptoms						
No	38.71 (6.60)	25.77–51.66	0.562	46.88 (5.18)	36.73–57.03	0.236
Yes	29.42 (5.35)	18.94–39.91		33.16 (4.85)	23.65–42.67	
ECOG						
<2	35.13 (4.72)	25.88–44.38	0.296	39.00 (4.58)	30.03–47.97	0.565
≥2	30.70 (7.71)	15.60–45.80		37.30 (6.88)	23.83–50.77	
LDH level						
≤245 U/L	33.39 (5.52)	22.58–44.21	0.813	39.30 (5.17)	29.17–49.44	0.652
>245 U/L	35.35 (6.19)	23.22–47.49		39.28 (5.63)	28.25–50.31	
IPI score						
0–1	35.25 (4.73)	25.97–44.53	0.356	39.08 (4.61)	30.04–48.11	0.616
2–5	31.20 (7.57)	16.36–46.04		37.80 (6.68)	24.70–50.90	
KPI score						
0–2	39.34 (5.13)	29.29–49.39	0.179	46.29 (4.46)	37.54–55.03	0.074
3–4	18.00 (3.99)	10.19–25.81		20.78 (3.29)	14.33–27.23	
Ann Arbor Stage						
I/II	33.76 (4.37)	25.20–42.33	0.380	37.41 (4.31)	28.97–45.86	0.471
III/IV	31.67 (6.79)	18.37–44.97		37.41 (6.04)	25.57–49.24	
E–PET						
Negative	41.52 (4.94)	31.83–51.20	0.014	47.00 (4.13)	38.91–55.09	0.019
Positive	14.67 (3.95)	6.92–22.42		19.10 (3.45)	12.33–25.87	

ECOG, Eastern Cooperative Oncology Group; IPI, International Prognostic Index; KPI, Korean prognostic index; E-PET, end-of-treatment PET/CT; PFS, progression-free survival; OS, overall survival.

**Table 7:** Multivariate analysis of factors predictive of progression-free survival and overall survival.

Risk Factor	PFS			Risk Factor	OS		
	Hazard ratio	95% CI	*p*		Hazard ratio	95% CI	*p*
Patients with B-PET (n = 47)				Patients with B-PET (n = 47)			
PET (SUVmax ≥ 15.1 vs. <15.1)	1.239	0.442–3.472	0.683	B-PET (SUVmax ≥ 15.1 vs. <15.1)	6.671	1.413–31.501	0.017
PET (MTV ≥ 16.11 vs. <16.11)	2.313	0.827–6.472	0.110	PET (MTV ≥ 16.11 vs. <16.11)	1.209	0.303–4.824	0.788
PET (TLG ≥ 45.90 vs. <45.90)	6.940	1.413–34.12	0.017	PET (TLG ≥ 45.90 vs. <45.90)	8.632	1.090–68.336	0.041
B symptoms (Yes vs. No)	1.010	0.341–2.991	0.986	Ann Arbor Stage (III/IV vs. I/II)	1.100	0.291–4.148	0.889
KPI score (3–4 vs. 0–2)	1.536	0.592–3.988	0.378	KPI score (3–4 vs. 0–2)	1.852	0.515–6.665	0.346
Patients with I-PET (n = 34)				Patients with I-PET (n = 34)			
I-PET (positive vs. negative)	5.355	1.646–17.424	0.005	I-PET (positive vs. negative)	5.596	1.159–27.015	0.032
B symptoms (Yes vs. No)	3.690	1.012–13.451	0.048	Ann Arbor Stage (III/IV vs. I/II)	4.333	1.081–17.371	0.038
LDH level (>245 vs. ≤245)	1.902	0.600–6.027	0.274	KPI score (3–4 vs. 0–2)	1.211	0.175–8.397	0.846
KPI score (3–4 vs. 0–2)	1.491	0.401–5.542	0.551				
Patients with E-PET (n = 28)				Patients with E-PET (n = 28)			
E-PET (positive vs. negative)	3.926	1.189–12.963	0.025	E-PET (positive vs. negative)	4.740	1.127–19.939	0.034
IPI score (2–5 vs. 0–1)	2.374	0.687–8.201	0.172	IPI score	1.124	0.233–5.421	0.884
KPI score (3–4 vs. 0–2)	1.044	0.250–4.366	0.953	KPI score (3–4 vs. 0–2)	2.135	0.457–9.978	0.335

B-PET, baseline PET/CT; I-PET, interim PET/CT; E-PET, end-of-treatment PET/CT; PFS, progression-free survival; OS, overall survival; SUVmax, maximum standardized uptake value; WBMTV, whole-body metabolic tumour volume; WBTLG, whole-body total lesion glycolysis; KPI, Korean prognostic index.

## References

[b1] Tse E. & Kwong Y. L. How I treat NK/T-cell lymphomas. Blood 121, 4997–5005 (2013). 23652805 10.1182/blood-2013-01-453233

[b2] Tse E. & Kwong Y. L. Nasal NK/T-cell lymphoma: RT, CT, or both. Blood 126, 1400–1401 (2015). 26384281 10.1182/blood-2015-07-655191

[b3] Sun J. . Distribution of lymphoid neoplasms in China: analysis of 4,638 cases according to the World Health Organization classification. Am J Clin Pathol. 138, 429–434 (2012). 22912361 10.1309/AJCP7YLTQPUSDQ5C

[b4] William B. M. & Armitage J. O. International analysis of the frequency and outcomes of NK/T-cell lymphomas. Best Pract Res Clin Haematol. 26, 23–32 (2013). 23768638 10.1016/j.beha.2013.04.003

[b5] Vose J., Armitage J. & Weisenburger D. International peripheral T-cell and natural killer/T-cell lymphoma study: pathology findings and clinical outcomes. J Clin Oncol. 26, 4124–4130 (2008). 18626005 10.1200/JCO.2008.16.4558

[b6] Au W. Y. . Clinical differences between nasal and extranasal natural killer/T-cell lymphoma: a study of 136 cases from the International Peripheral T-Cell Lymphoma Project. Blood 113, 3931–3937 (2009). 19029440 10.1182/blood-2008-10-185256

[b7] Elstrom R. L., Leonard J. P., Coleman M. & Brown R. K. Combined PET and low-dose, noncontrast CT scanning obviates the need for additional diagnostic contrast-enhanced CT scans in patients undergoing staging or restaging for lymphoma. Ann Oncol. 19, 1770–1773 (2008). 18550578 10.1093/annonc/mdn282 PMC2735066

[b8] Raanani P. . Is CT scan still necessary for staging in Hodgkin and non-Hodgkin lymphoma patients in the PET/CT era? Ann Oncol. 17, 117–122 (2006). 16192294 10.1093/annonc/mdj024

[b9] Luminari S. . The use of FDG-PET in the initial staging of 142 patients with follicular lymphoma: a retrospective study from the FOLL05 randomized trial of the Fondazione Italiana Linfomi. Ann Oncol. 24, 2108–2112 (2013). 23585513 10.1093/annonc/mdt137

[b10] Biggi A. . International validation study for interim PET in ABVD-treated, advanced-stage hodgkin lymphoma: interpretation criteria and concordance rate among reviewers. J Nucl Med. 54, 683–690 (2013). 23516309 10.2967/jnumed.112.110890

[b11] Safar V. . Interim [18F] fluorodeoxyglucose positron emission tomography scan in diffuse large B-cell lymphoma treated with anthracycline-based chemotherapy plus rituximab. J Clin Oncol. 30, 184–190 (2012). 22162590 10.1200/JCO.2011.38.2648

[b12] Hutchings M. . Position emission tomography with or without computed tomography in the primary staging of Hodgkin’s lymphoma. Haematologica. 91, 482–489 (2006). 16585015

[b13] El-Galaly T. C. . Routine bone marrow biopsy has little or no therapeutic consequence for positron emission tomography/computed tomography-staged treatment-naive patients with Hodgkin lymphoma. J Clin Oncol. 30, 4508–4514 (2012). 23150698 10.1200/JCO.2012.42.4036

[b14] Khan A. B. . PET-CT staging of DLBCL accurately identifies and provides new insight into the clinical significance of bone marrow involvement. Blood 122, 61–67 (2013). 23660958 10.1182/blood-2012-12-473389

[b15] Jiang C. . Assessment of the prognostic capacity of pretreatment, interim, and post-therapy (18) F-FDG PET/CT in extranodal natural killer/T-cell lymphoma, nasal type. Ann Nucl Med. 29, 442–451 (2015). 25801633 10.1007/s12149-015-0964-8

[b16] Kim C. . Prognostic value of whole-body metabolic tumour volume and total lesion glycolysis measured on 18F-FDG PET/CT in patients with extranodal NK/T-cell lymphoma. European Journal of Nuclear Medicine and Molecular Imaging 40, 1321–1329 (2013). 23674211 10.1007/s00259-013-2443-6

[b17] Khong P. L., Pang C. B., Liang R., Kwong Y. L. & Au W. Y. Fluorine-18 fluorodeoxyglucose positron emission tomography in mature T-cell and natural killer cell malignancies. Ann Hematol. 87, 613–621 (2008). 18509641 10.1007/s00277-008-0494-8

[b18] Khong P. L., Huang B., Lee E. Y., Chan W. K. & Kwong Y. L. Midtreatment (18) F-FDG PET/CT scan for early response assessment of SMILE therapy in natural killer/T-cell lymphoma: a prospective study from a single center. J Nucl Med. 55, 911–916 (2014). 24819420 10.2967/jnumed.113.131946

[b19] Li Y. J. . Prognostic value of interim and posttherapy 18F-FDG PET/CT in patients with mature T-cell and natural killer cell lymphomas. J Nucl Med. 54, 507–515 (2013). 23397008 10.2967/jnumed.112.110262

[b20] Sabattini E., Bacci F., Sagramoso C. & Pileri S. A. WHO classification of tumours of haematopoietic and lymphoid tissues in 2008: an overview. Pathologica. 102, 83–87 (2010). 21171509

[b21] Meignan M., Gallamini A., Meignan M., Gallamini A. & Haioun C. Report on the first international workshop on interim-PET-scan in lymphoma. Leuk Lymphoma. 50, 1257–1260 (2009). 19544140 10.1080/10428190903040048

[b22] Barrington S. F. . Role of imaging in the staging and response assessment of lymphoma: consensus of the International Conference on Malignant Lymphomas Imaging Working Group. J Clin Oncol. 32, 3048–3058 (2014). 25113771 10.1200/JCO.2013.53.5229 PMC5015423

[b23] Chan W. K. . Metabolic activity measured by F-18 FDG PET in natural killer-cell lymphoma compared to aggressive B- and T-cell lymphomas. Clin Nucl Med. 35, 571–575 (2010). 20631501 10.1097/RLU.0b013e3181e4dcbf

[b24] Moon S. H. . The role of 18F-FDG PET/CT for initial staging of nasal type natural killer/T-cell lymphoma: a comparison with conventional staging methods. J Nucl Med. 54, 1039–1044 (2013). 23658217 10.2967/jnumed.112.113399

[b25] Zhou X. . Utility of PET/CT in the diagnosis and staging of extranodal natural killer/T-cell lymphoma: a systematic review and meta-analysis. Medicine (Baltimore). 93, e258 (2014). 25526450 10.1097/MD.0000000000000258 PMC4603121

[b26] Suh C. . Prognostic value of tumor 18F-FDG uptake in patients with untreated extranodal natural killer/T-cell lymphomas of the head and neck. J Nucl Med. 49, 1783–1789 (2008). 18927319 10.2967/jnumed.108.053355

[b27] Xie M. . Predictive value of F-18 FDG PET/CT quantization parameters for progression-free survival in patients with diffuse large B-cell lymphoma. Hematology 21, 99–105 (2016). 26183456 10.1179/1607845415Y.0000000033

[b28] Zhang L. J. . The significance of 18F-FDG PET/CT in secondary hemophagocytic lymphohistiocytosis. J Hematol Oncol. 5, 40 (2012). 22824539 10.1186/1756-8722-5-40 PMC3461421

[b29] Kim J. . Clinical implication of F-18 FDG PET/CT in patients with secondary hemophagocytic lymphohistiocytosis. Ann Hematol. 93, 661–667 (2014). 24061788 10.1007/s00277-013-1906-y

[b30] Chim C. S. . Primary nasal natural killer cell lymphoma: long-term treatment outcome and relationship with the International Prognostic Index. Blood 103, 216–221 (2004). 12933580 10.1182/blood-2003-05-1401

[b31] Kim T. M. & Heo D. S. Extranodal NK/T-cell lymphoma, nasal type: new staging system and treatment strategies. Cancer Sci. 100, 2242–2248 (2009). 19758393 10.1111/j.1349-7006.2009.01319.x PMC11159079

[b32] Lee J. . Extranodal natural killer T-cell lymphoma, nasal-type: a prognostic model from a retrospective multicenter study. J Clin Oncol. 24, 612–618 (2006). 16380410 10.1200/JCO.2005.04.1384

[b33] Zinzani P. L. . Midtreatment 18F-fluorodeoxyglucose positron-emission tomography in aggressive non-Hodgkin lymphoma. Cancer 117, 1010–1018 (2011). 20960498 10.1002/cncr.25579

[b34] Huntington S. F., Nasta S. D., Schuster S. J., Doshi J. A. & Svoboda J. Utility of interim and end-of-treatment (18F)-fluorodeoxyglucose positron emission tomography–computed tomography in frontline therapy of patients with diffuse large B-cell lymphoma. Leukemia & Lymphoma. 56, 2579–2584 (2015). 25629993 10.3109/10428194.2015.1007506

[b35] Cheson B. D. . Revised response criteria for malignant lymphoma. J Clin Oncol. 25, 579–586 (2007). 17242396 10.1200/JCO.2006.09.2403

[b36] Juweid M. E. . Use of positron emission tomography for response assessment of lymphoma: consensus of the Imaging Subcommittee of International Harmonization Project in Lymphoma. J Clin Oncol. 25, 571–578 (2007). 17242397 10.1200/JCO.2006.08.2305

[b37] Kwong Y. L. . SMILE for natural killer/T-cell lymphoma: analysis of safety and efficacy from the Asia Lymphoma Study Group. Blood 120, 2973–2980 (2012). 22919026 10.1182/blood-2012-05-431460

[b38] Yamaguchi M. . Phase II study of SMILE chemotherapy for newly diagnosed stage IV, relapsed, or refractory extranodal natural killer (NK)/T-cell lymphoma, nasal type: the NK-Cell Tumor Study Group study. J Clin Oncol. 29, 4410–4416 (2011). 21990393 10.1200/JCO.2011.35.6287

[b39] Wang L. . First-line combination of gemcitabine, oxaliplatin, and L-asparaginase (GELOX) followed by involved-field radiation therapy for patients with stage IE/IIE extranodal natural killer/T-cell lymphoma. Cancer. 119, 348–355 (2013). 22811078 10.1002/cncr.27752

[b40] Lin N. . A prospective phase II study of L-asparaginase- CHOP plus radiation in newly diagnosed extranodal NK/T-cell lymphoma, nasal type. J Hematol Oncol. 6, 44 (2013). 23816178 10.1186/1756-8722-6-44 PMC3734195

[b41] Li L. . Efficacy of a pegaspargase-based regimen in the treatment of newly-diagnosed extranodal natural killer/T-cell lymphoma. Neoplasma 61, 225–232 (2014). 24299319 10.4149/neo_2014_029

[b42] Zhou Z. . Effectiveness of gemcitabine, pegaspargase, cisplatin, and dexamethasone (DDGP) combination chemotherapy in the treatment of relapsed/refractory extranodal NK/T cell lymphoma: a retrospective study of 17 patients. Ann Hematol. 93, 1889–1894 (2014). 24923454 10.1007/s00277-014-2136-7

[b43] Barnes J. A. . End-of-treatment but not interim PET scan predicts outcome in nonbulky limited-stage Hodgkin’s lymphoma. Ann Oncol. 22, 910–915 (2011). 20952598 10.1093/annonc/mdq549

[b44] Cerci J. J. . Cost effectiveness of positron emission tomography in patients with Hodgkin’s lymphoma in unconfirmed complete remission or partial remission after first-line therapy. J Clin Oncol. 28, 1415–1421 (2010). 20142591 10.1200/JCO.2009.25.4367

[b45] Pregno P. . Interim 18-FDG-PET/CT failed to predict the outcome in diffuse large B-cell lymphoma patients treated at the diagnosis with rituximab-CHOP. Blood. 119, 2066–2073 (2012). 22234681 10.1182/blood-2011-06-359943

[b46] Kim S. J. . Risk stratification on the basis of Deauville score on PET-CT and the presence of Epstein-Barr virus DNA after completion of primary treatment for extranodal natural killer/T-cell lymphoma, nasal type: a multicentre, retrospective analysis. Lancet Haematol. 2, e66–e74 (2015). 26687611 10.1016/S2352-3026(15)00002-2

[b47] Jiang C. . The Deauville 5-point scale improves the prognostic value of interim FDG PET/CT in extranodal natural killer/T-cell lymphoma. Clin Nucl Med. 40, 767–773 (2015). 26164182 10.1097/RLU.0000000000000892

[b48] Moskowitz C. H. . Risk-adapted dose-dense immunochemotherapy determined by interim FDG-PET in Advanced-stage diffuse large B-Cell lymphoma. J Clin Oncol. 28, 1896–1903 (2010). 20212248 10.1200/JCO.2009.26.5942 PMC3651601

